# Rates and Factors Associated with Major Modifications to First-Line Combination Antiretroviral Therapy: Results from the Asia-Pacific Region

**DOI:** 10.1371/journal.pone.0064902

**Published:** 2013-06-28

**Authors:** Stephen Wright, Mark A. Boyd, Evy Yunihastuti, Matthew Law, Thira Sirisanthana, Jennifer Hoy, Sanjay Pujari, Man Po Lee, Kathy Petoumenos, on behalf of the International Epidemiologic Databases to Evaluate AIDS (IeDEA) Asia-Pacific HIV Observational Database (APHOD)

**Affiliations:** 1 Kirby Institute, Sydney, Australia; 2 St. Vincent’s Hospital, Sydney, Australia; 3 Cipto Mangunkusumo General Hospital, Jakarta Pusat, Indonesia; 4 Chiang Mai University, Chiang Mai, Thailand; 5 The Alfred Hospital, Melbourne, Australia; 6 Monash University, Melbourne, Australia; 7 Institute of Infectious Diseases, Pune, India; 8 Queen Elizabeth Hospital, Kowloon, Hong Kong; McGill University AIDS Centre, Canada

## Abstract

**Background:**

In the Asia-Pacific region many countries have adopted the WHO’s public health approach to HIV care and treatment. We performed exploratory analyses of the factors associated with first major modification to first-line combination antiretroviral therapy (ART) in resource-rich and resource-limited countries in the region.

**Methods:**

We selected treatment naive HIV-positive adults from the Australian HIV Observational Database (AHOD) and the TREAT Asia HIV Observational Database (TAHOD). We dichotomised each country’s per capita income into high/upper-middle (T-H) and lower-middle/low (T-L). Survival methods stratified by income were used to explore time to first major modification of first-line ART and associated factors. We defined a treatment modification as either initiation of a new class of antiretroviral (ARV) or a substitution of two or more ARV agents from within the same ARV class.

**Results:**

A total of 4250 patients had 961 major modifications to first-line ART in the first five years of therapy. The cumulative incidence (95% CI) of treatment modification was 0.48 (0.44–0.52), 0.33 (0.30–0.36) and 0.21 (0.18–0.23) for AHOD, T-H and T-L respectively. We found no strong associations between typical patient characteristic factors and rates of treatment modification. In AHOD, relative to sites that monitor twice-yearly (both CD4 and HIV RNA-VL), quarterly monitoring corresponded with a doubling of the rate of treatment modifications. In T-H, relative to sites that monitor once-yearly (both CD4 and HIV RNA-VL), monitoring twice-yearly corresponded to a 1.8 factor increase in treatment modifications. In T-L, no sites on average monitored both CD4 & HIV RNA-VL concurrently once-yearly. We found no differences in rates of modifications for once- or twice-yearly CD4 count monitoring.

**Conclusions:**

Low-income countries tended to have lower rates of major modifications made to first-line ART compared to higher-income countries. In higher-income countries, an increased rate of RNA-VL monitoring was associated with increased modifications to first-line ART.

## Introduction

The introduction of combination antiretroviral therapy (ART) has dramatically changed the management of HIV infection. Combination ART can suppress plasma HIV replication to levels below the level of detection of highly sensitive contemporary assays, allowing reconstitution of the immune system and concomitant protection against AIDS diseases and mortality [1], [2]. Since the introduction of zidovudine (AZT) in 1987, ART has evolved rapidly over time with multiple agents now licensed for use [3], [4]. In high-income countries where access to ART is widely available, an effective regimen usually consists of a combination of antiretrovirals selected from five classes, where each class targets independent viral replication processes. The availability of five classes of ART gives prescribers a variety of potential initial as well as second- and third-line treatment options that afford full virological suppression [5]. In high-income countries, initial ART regimens are individualized in order to minimise toxicity and adverse events, and maximise tolerability and efficacy of treatment. This is done to facilitate the chance of good adherence and thereby successful long-term HIV treatment outcomes.

In low- and middle-income countries the availability of ART is generally more restricted, limiting prescription to a narrower selection of ART combinations. Monitoring of disease, including HIV RNA viral load monitoring, is also restricted in many of these settings as is the widespread availability of expert medical care. In response, the World Health Organization (WHO) has recommended that access to HIV care including ART in such settings be facilitated by a public health based approach. This approach provides access to simple, affordable and well-studied ART regimens and disease monitoring schedules that allow for adequate care in the absence of sophisticated and expensive medical technologies [6].

The Asia-Pacific region has a large burden of HIV with estimates of around 4.9 million persons living with HIV/AIDS [7]. Over 85% of this burden is carried by India, China, Thailand, Indonesia and Vietnam [7]. The scale-up of ART in the Asia-Pacific region has increased substantially over recent years with most countries within the region now having widespread access to first-line ART, but only minimal second-line therapy options [8]. Most countries within the region have adopted a public health approach-based treatment strategy aligned with the WHO recommendations [6]. However, in this politically and economically diverse region, a number of approaches to the management of HIV exist. Different strategies have been adopted according to a range of considerations including HIV monitoring, ART dosing, and were formed partly on lack of evidence as to best practice [9].

In the absence of a cure, ART is considered to be a life-long means of achieving virological control. Numerous studies have shown that ART prolongs and improves the quality of life of a person living with HIV. In some settings, survival of people living with HIV may be similar to that observed in HIV-negative populations [10]–[12]. Poor adherence to the appropriate ART dosing schedule selects for drug resistance and can shorten the regimens durability [13]. Because of the critical importance of achieving and maintaining virological control, prescribers in collaboration with their patients may adapt and change ART regimens if individuals are experiencing barriers to adequate adherence. This decision to modify treatment is an important consideration as there are currently only a finite number of active ART combinations available for life-long treatment [14].

There are minimal data published on the rates of switching or modifying first-line ART for any reason in the Asia-Pacific region. The objective of this analysis was to explore the differences between resource rich and resource limited countries rates of major modifications made to first-line ART. Specifically we seek to further describe by income group, the first ART regimens initiated, frequency of HIV disease monitoring, and factors associated with major modifications to first-line ART regimens.

## Methodology

### Data Collection

The Asia-Pacific HIV Observational Database (APHOD) consists of two adult and one pediatric cohort and is part of the International Epidemiological Database Evaluating AIDS (IeDEA) collaboration. This analysis utilises data from the two adult cohorts in the Asia-Pacific region; the Australian HIV Observational Database (AHOD) and the TREAT Asia HIV Observational Database (TAHOD). APHOD data are collected twice annually (March & September) on a core set of demographic and clinical variables including information to assess and monitor patterns of ART, treatment efficacy, patterns of toxicity, markers of disease stage, and to monitor HIV-related and non-HIV-related causes of death. Data collection methodology for each cohort and baseline clinical characteristics have been described elsewhere [15], [16]. Data are transferred electronically to The Kirby Institute, Sydney and all data are subjected to internal quality control and assurance procedures. This analysis is based on all data collated as of 31 March 2011 for AHOD and 30 September 2011 for TAHOD.

Ethics and study governance approval for AHOD was granted by the University of New South Wales Human Research Ethics Committee, and site-specific study governance granted by their nominated institutional review boards. Patient’s written and informed consent was obtained at time of enrolment. Ethics approval was granted for TAHOD by the University of New South Wales Human Research Ethics Committee. Sites specific study governance was granted by site-relevant institutional review boards. Written informed consent was not sought in TAHOD unless required by a site’s local institutional review board. Informed consent was waived at some sites as information is collected via an anonymous case report form. All study procedures were developed in accordance with the revised 1975 Helsinki Declaration.

We included all patients with a known starting date of combination ART who had not previously received mono/duo ART (treatment naive). We defined initial ART as two nucleos(t)ide reverse transcriptase inhibitor (N(t)RTI) anchored with one of the following; non-nucleoside reverse transcriptase inhibitor (NNRTI), protease inhibitor (PI), or integrase inhibitor (II). To reduce the reliance on treatment records captured prior to patient enrolment and to ensure our analysis was based largely on treatment records captured after enrolment, we further restricted our analysis population to patients who initiated ART after or ≤two years prior to cohort enrolment. We excluded patients who reported an undetectable plasma HIV RNA viral load (RNA-VL) measurement (defined as <400 copies/ml) prior to the reported ART initiation date.

### Statistical Analysis

For comparative purposes we classified countries participating in TAHOD into a dichotomous income variable based on the World Bank’s per capita 2009 gross national income (GNI) estimates [17]. We defined high- to upper middle-income as per capita GNI>$3945 and lower middle- to low-income as per capita GNI<$3946. We summarised typical patient characteristics by income grouping and evaluated the frequency of HIV disease monitoring.

The primary endpoint for this analysis was time until first major first-line treatment modification, defined as either initiating a new class of antiretroviral or a substitution of two or more of the original antiretroviral agents with different agents from within the same ART class. Patients were censored at five years of follow-up after initiating ART. We defined a patient as lost to follow-up if they had not presented to care for at least one year prior to the last site’s data transfer date. Patients who died were considered to have completed follow-up and were censored at their date of death. We recorded the primary reason for ART modification as identified by the treating nurse or physician. Reasons for modifying an ART regimen were aggregated into four broad categories- treatment failure (clinical, immunological, virological), adverse event or toxicity, patient or physician decision (including drug-drug interaction switches) and unknown/not reported.

We tabulated by income grouping (AHOD, TAHOD-High, TAHOD-Low) the number of individual antiretroviral agents used at ART initiation, class of anchor agent used in the first-line ART regimen, reported reason for ART modification, and ART regimen switched-to. We used competing risk methods (as described by Fine and Gray [18]) to estimate the cumulative incidence of patients that made a major modification to their first-line ART regimen. We used multivariable Cox proportional hazards methods to evaluate any association between patient characteristics and rates of major modification to ART. Factors evaluated included sex, age at ART initiation, HIV exposure category (men who have sex with men, injecting drug user, heterosexual contact and other), hepatitis B virus (HBV) and hepatitis C virus (HCV) seropositivity, year of ART initiation (1996–2004 or 2005–2011), pre-ART AIDS-defining illness, pre-ART CD4 count (0–199, 200–349, 350–500, >500 cells/µL or unknown), pre-ART HIV RNA-VL (1–10^4^, 10^4^–10^6^, >10^6^ copies/ml and unknown) and an indicator for site resourcing (see below). Multivariable models were run separately on subsets of data split by income grouping. For comparative purposes we applied the same a priori defined covariate models and used the same reference level for each categorical factor across each model.

Each country within its respective income grouping has different levels of resources allocated to manage HIV. Furthermore, within each country, individual treating sites can have differing capacities to provide HIV care and treatment (e.g. urban vs rural). To evaluate associations between site resourcing and rates of major modifications to ART, we used a proxy indicator variable for site resourcing. We defined site resourcing as a function of the average proportion of patients having “x” yearly, routine HIV monitoring laboratory measurements (RNA-VL and CD4 cell counts). To assign this indicator we first calculated for each site the proportion of patients that had on average in a year- one, two, three or four CD4 cell counts measured along with zero, one, two, three or four RNA-VL measurements. Using a median cutoff, we assigned for each site the highest number of measurements for which the average proportion of patients was greater than 50%. For example, if a site on average had 78% of its patients having three or more CD4 cell count measurement per year, but only 45% for four or more CD4 cell count measurements per year, then it would be assigned an average of three CD4 cell count measurements per year. Similarly, for RNA-VL, if the same site on average had 89% of its patients having two RNA-VL measurement per year, but only 36% for three RNA-VL measurements per year, then it would be assigned an average of two RNA-VL measurements per year. The two monitoring schedules were then concatenated to form the resourcing variable – i.e. 3CD4::2RNA-VL. Using a 50% cutoff point for the classification of site resourcing has the interpretation- *more than half of the patients at the site had 3 or more CD4 cell counts measurements and 2 or more HIV RNA-VL measurements per year*. It was not a requirement that CD4 cell counts and RNA-VL had to be measured on the same date.

## Results

Of the 3378 AHOD patients enrolled by 31 March 2011, 945 (28%) satisfied the analysis eligibility criteria. Of the AHOD patients excluded from the analysis, 2111 (62%) were not included due to timing of ART initiation (ART initiation date >2 years prior to enrolment) and 158 (5%) were excluded as they received mono/duo therapy prior to first-line ART. Similarly for TAHOD, of the 5988 TAHOD patients enrolled by 30 September 2011, 3305 (55%) satisfied the analysis eligibility criteria. Of the TAHOD patients excluded from the analysis, 2444 (41%) were not included due to the timing of ART initiation and 115 (2%) were excluded for prior mono/duo therapy exposure. The mean time followed on ART for AHOD and TAHOD-High was five years compared to approximately three years for TAHOD-Low. Similar rates (95% confidence intervals) of patients lost to follow up are reported for AHOD: 4.9 (4.3–5.6) per 100 patient years and TAHOD-High: 4.6 (4.1–5.1) per 100 patient-years. TAHOD-Low had a slightly higher rate, 7 (6.4–7.7) per 100 patient-years. The crude mortality rate for AHOD was 0.82 (0.61–1.12) per 100 patient years, for TAHOD-High: 0.92 (0.71–1.19) per 100 patient years and for TAHOD-Low: 1.64 (1.34–2.00) per 100 patient years.

The estimated rate of a major modification in AHOD was 14.7 (13.3–16.4) per 100 person-years (Table 1). The highest major modification rate for TAHOD-High was in Singapore, 15.7 (11.3–21.8) per 100 person-years and the lowest in Hong Kong, 5.7 (3.5–9.2) per 100 person-years. Similarly for TAHOD-Low, the highest rate was in India, 6.7 (5.5–8.1) per 100 person-years and lowest in Vietnam, 3.3 (2.0–5.5) per 100 person-years. The average patient age at ART initiation in AHOD was 41 years compared to 38 years for TAHOD-High and 35 years old for TAHOD-Low (Table 2). AHOD HIV exposure is predominately through homosexual contact as opposed to TAHOD in which the majority reported transmission is through heterosexual contact. The proportion of HIV exposure through injecting drug use (IDU) was highest in TAHOD-Low (16%) vs. TAHOD-High (3%) or AHOD (5%). There were differences for HBV surface antigen positivity across AHOD (5%) and TAHOD (7–14%). TAHOD-High/Low reported more AIDS-defining illnesses, 59–65% of patients, compared to AHOD- 16%. Of those AIDS-defining illnesses, timing of the event, pre-ART or post-ART, were similar. Mean pre-ART CD4 cell count was approximately 2-fold higher in AHOD compared to TAHOD-High/Low (300 vs. 150 cells/µL). Pre-ART RNA-VL was similar across the income groups. The proportion of patients initiating ART in more recent periods, 2005–2011, was highest in TAHOD-Low (75%) compared to AHOD (43%) and TAHOD-High (42%). The estimated median (q1–q3 quartiles) number of CD4 cell measurements per year per patient are AHOD: 3 (2–4), TAHOD-High: 2 (1–2), TAHOD-Low: 1 (1–2). The estimated median number of RNA-VL measurements were as follows: AHOD: 3 (2–4), TAHOD-High: 2 (1–2), TAHOD-Low: 0 (0–1).

**Table 1:** Crude rate of first major modification to ART and average per patient per year HIV monitoring (CD4 cell count and HIV RNA-VL) by country.

					*Percentage of cohort that have 1 or 2 CD4/RNA measurement per year*			
Income grouping	Country	Start of cohort	No. Patients (switches)	Rate of switch (95% CI)	1:CD4 (%)	1:RNA (%)	2:CD4 (%)	2:RNA (%)
AHOD	Australia	1999	945 (350)	14.7 (13.3–16.4)	*93*	*91*	*79*	*78*
High/Upper	Singapore	2003	87 (36)	15.7 (11.3–21.8)	*96*	*49*	*82*	*15*
Middle	Taiwan	2003	175 (67)	12.6 (9.9–16)	*89*	*87*	*65*	*65*
	Malaysia	2003	155 (46)	9.3 (6.9–12.4)	*92*	*86*	*56*	*48*
	South Korea	2007	113 (24)	9.1 (6.1–13.5)	*99*	*98*	*79*	*79*
	Japan	2005	180 (48)	8.1 (6.1–10.7)	*95*	*95*	*73*	*73*
	Thailand	2003	471 (114)	7.1 (5.9–8.5)	*92*	*71*	*55*	*18*
	Hong Kong SAR	2003	79 (17)	5.7 (3.5–9.2)	*100*	*98*	*95*	*62*
Lower middle/	India	2003	523 (104)	6.7 (5.5–8.1)	*85*	*28*	*46*	*15*
Low	China	2003	257 (35)	5.5 (4–7.7)	*87*	*45*	*46*	*16*
	Philippines	2003	175 (31)	5.4 (3.8–7.7)	*71*	*6*	*23*	*0*
	Cambodia	2005	208 (36)	4.9 (3.6–6.8)	*100*	*12*	*72*	*1*
	Indonesia	2004	392 (38)	4 (2.9–5.5)	*87*	*18*	*41*	*2*
	Vietnam	2010	490 (15)	3.3 (2–5.5)	*99*	*18*	*45*	*6*

**Table 2:** Patient demographics by country income grouping.

	AHOD	TAHOD	TAHOD
	(n = 945)	High Income	Low Income
		(n = 1260)	(n = 2045)
**Sex**	***N (%)***	***N (%)***	***N (%)***
Male	891 (94)	951 (75)	1434 (70)
Female	54 (4)	309 (25)	611 (30)
**Age at ART initiation (Years)**			
less than 30	121 (13)	273 (22)	590 (29)
30 to 50	646 (68)	829 (66)	1313 (64)
greater than 50	178 (19)	158 (13)	142 (7)
mean	41.1	38.1	35.1
median (interquartile range)	40.3 (33.4–47.5)	36.2 (30.9–43.4)	33.8 (29.1–39.5)
**AIDS**			
None	793 (84)	511 (41)	709 (35)
Yes	152 (16)	749 (59)	1336 (65)
AIDS illness pre-ART	50 (33)	173 (23)	343 (26)
AIDS illness post-ART	102 (67)	576 (77)	993 (74)
**Exposure**			
Homosexual	644 (68)	392 (31)	151 (7)
Heterosexual	120 (13)	736 (58)	1345 (66)
IDU	50 (5)	34 (3)	319 (16)
Other	131 (14)	98 (8)	230 (11)
**HBV**			
Negative	721 (76)	884 (70)	1202 (59)
Positive	36 (4)	175 (14)	134 (7)
Not Tested	188 (20)	201 (16)	709 (35)
**HCV**			
Negative	737 (78)	887 (70)	769 (38)
Positive	80 (8)	78 (6)	384 (19)
Not Tested	128 (14)	295 (23)	892 (44)
**CD4 cell count at ART initiation** *			
mean (cells/µL)	305	150	128
median (interquartile range)	271 (159–400)	125 (34–227)	110 (38–199)
**Log RNA at ART initiation** *			
mean (copies/ml)	4.85	4.7	4.96
median (interquartile range)	4.96 (4.44–5.35)	4.73 (4.15–5.30)	5.0 (4.56–5.57)
**Year ART Initiation**			
1996 to 2004	499 (53)	728 (58)	507 (25)
2005 to 2011	446 (47)	532 (42)	1538 (75)
**Time followed on ART**			
mean (years)	5.3	5.1	3.1
median (interquartile range)	3.8 (1.8–8.8)	5.5 (2.9–7.5)	2.4 (0.9–5.1)
**CD4 measurements (per year per person)**			
mean	3.4	1.9	1.7
median (interquartile range)	3 (2–4)	2 (1–2)	1 (1–2)
**RNA measurements (per year per person)**			
Mean	3.3	1.4	0.6
median (interquartile range)	3 (2–4)	1 (1–2)	0 (0–1)

* Closest record to ART initiation within a window of 6 month prior to ART and 1 week following ART initiation.

**Figure 1:**
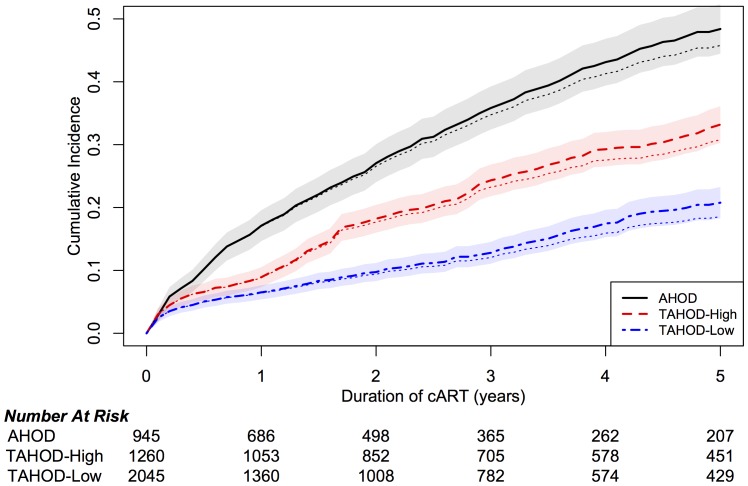
Time to first major modification of ART (any reason) by country income grouping. Cumulative Incidence are adjusted for competing risk of death (solid line) with mean 95% confidence bands. Sensitivity analysis (dashed line) adjusts for competing risk of death or lost to follow-up.

Table 3 presents by income group the distribution of anchor agent used in first-line ART initiated and the regimen switch-to following modification. The proportions of reasons given for treatment modification by income group are also presented in Table 3. The rate of major modification made to first-line ART by income group adjusted for competing risk of death (dashed line - competing risk of death and lost to follow-up) are presented, along with an at-risk population table in Figure 1. After five years of ART, adjusting for the competing risk of death, the cumulative incidence (95% CI) of first-line treatment modifications was 0.48 (0.44–0.52) for AHOD, 0.33 (0.30–0.36) for TAHOD-High and 0.21 (0.18–0.23) for TAHOD-Low.

**Table 3:** Antiretroviral agents available for first-line ART, ART anchor agent, reported reason for major modification and selected switched-to ART regimen by country income grouping.

	AHOD	TAHOD	TAHOD
	N (%)	High Income	Low Income
		N (%)	N (%)
**Number of different ARV agents used for initial ART regimen** *			
N(t)RTI	9	7	7
NNRTI	2	2	2
PI	8	9	5
II	1	1	0
**Initial ART regimen**			
2 N(t)RTI+NNRTI	574 (61)	852 (68)	1976 (97)
2 N(t)RTI+PI	279 (30)	364 (29)	67 (3)
2 N(t)RTI+II	25 (3)	6 (0)	0 (0)
Other (non-standard, e.g. 3 N(t)RTI)	67 (7)	38 (3)	<5 (0)
**Number of switches from initial ART regimen**	350	352	259
*Reported reason for modification*			
Treatment failure	47 (19)	45 (13)	73 (29)
Adverse event/toxicity	97 (38)	141 (41)	107 (42)
Patient decision/Physician decision	110 (43)	162 (47)	76 (30)
Not reported	96	<5	<5
**Modified ART regimen**			
*Initial ART: 2 N(t)RTI+NNRTI*			
2 N(t)RTI+NNRTI	48 (30)	67 (34)	85 (35)
2 N(t)RTI+PI	75 (47)	69 (35)	66 (27)
2 N(t)RTI+II	6 (4)	0 (0)	0 (0)
Other (non-standard)	29 (18)	63 (32)	93 (38)
*Initial ART: 2 N(t)RTI+PI*			
2 N(t)RTI+PI	42 (27)	80 (65)	<5 (10)
2 N(t)RTI+NNRTI	64 (41)	30 (24)	9 (65)
2 N(t)RTI+II	9 (6)	<5 (5)	0 (0)
Other (non-standard)	41 (26)	8 (6)	<5 (25)

* Individual ARV agents with each class where less than 5 people initiated where excluded from the total count.

N(t)RTI = Nucleos(t)ide Reverse Transcriptase Inhibitor, NNRTI = Non-Nucleoside Reverse Transcriptase Inhibitor, PI = Protease Inhibitors, II = Integrase Inhibitor.

**Figure 2:**
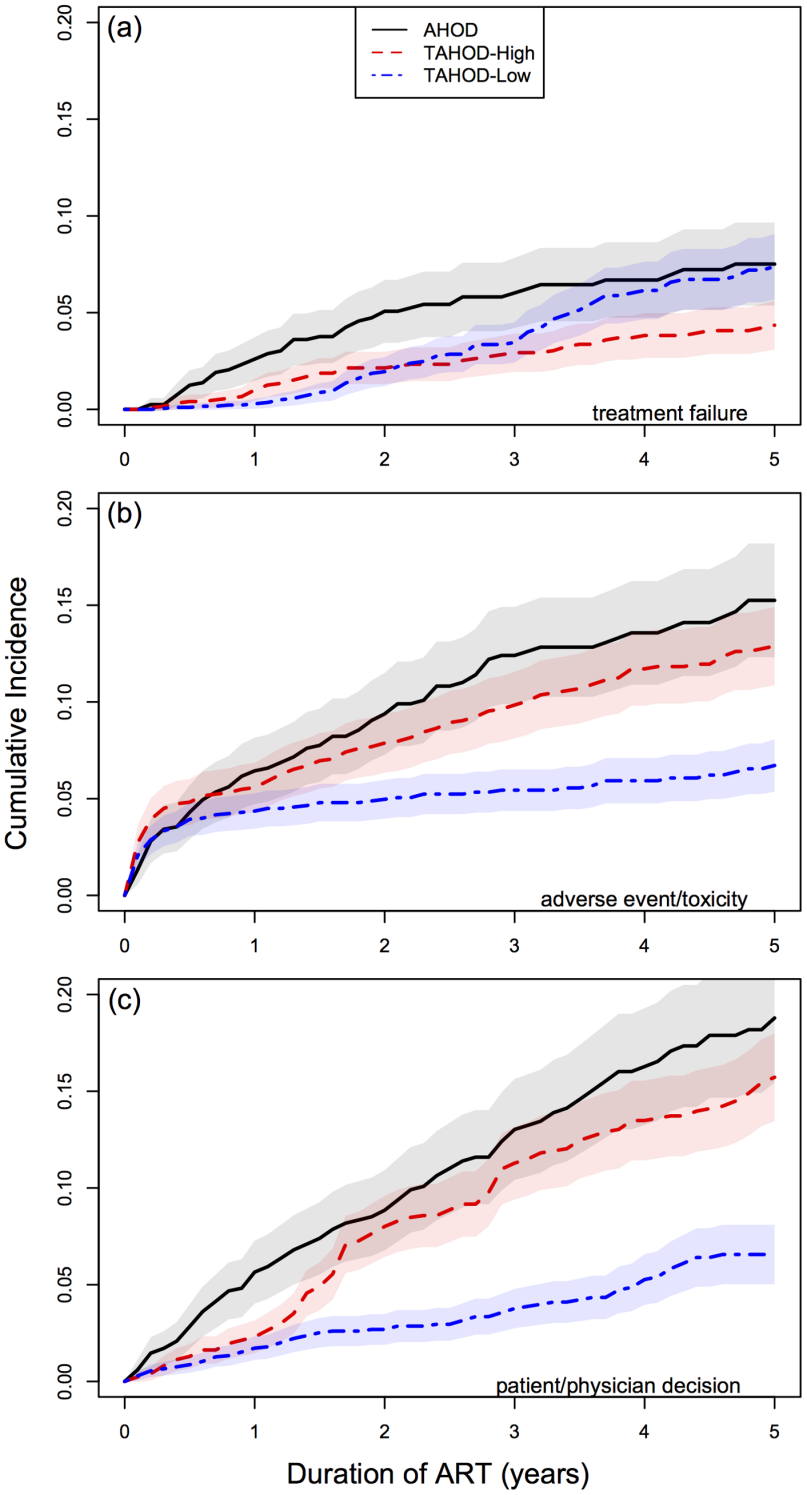
Time to first major modification of ART by country income grouping stratified by reported reason for modification. Panel (a) - reported treatment failure, Panel - (b) adverse event/toxicity, Panel (c) - patient/physician decision. Cumulative Incidence are adjusted for competing risk of death and competing reasons for modification. Shaded bands are mean 95% confidence bands.

The cumulative incidence for treatment modification adjusted for competing risk of death and competing reason for modification are presented in Figure 2. Table 4 outlines clinical factors and associations of rates of major modifications to first-line ART by income grouping. Multivariable estimated hazard ratios by income grouping were similar in relative magnitude and direction for most covariates. The majority of covariates including sex, age at ART initiation, HIV exposure, HBV serostatus, pre-ART AIDS-defining Illness, pre-ART CD4 and pre-ART RNA-VL, were not significantly associated with major treatment modifications. HCV positive serostatus and more recent periods of ART initiation were indicative of reduced rates of treatment modification across all income grouping models.

**Table 4:** Predictors of major first-line modification by income grouping.

	AHOD		TAHOD-High		TAHOD-Low	
	Hazard Ratio	p	Hazard Ratio	p	Hazard Ratio	p
**Sex**						
Male	1.00		1.00		1.00	
Female	0.92 (0.55–1.55)	0.75	0.93 (0.7–1.25)	0.64	0.76 (0.56–1.03)	0.08
**Age at ART initiation (years)**						
<30	1.00		1.00		1.00	
30–50	1.07 (0.76–1.49)	0.71	0.79 (0.61–1.03)	0.08	1.04 (0.77–1.4)	0.81
>50	1.14 (0.76–1.71)	0.52	0.87 (0.6–1.25)	0.45	0.81 (0.46–1.44)	0.47
**Exposure**						
Homosexual	1.00		1.00		1.00	
Heterosexual	0.92 (0.63–1.34)	0.67	1.34 (0.97–1.86)	0.08	1.17 (0.69–1.98)	0.56
IDU	1.29 (0.81–2.07)	0.28	1.57 (0.81–3.05)	0.18	1.16 (0.58–2.33)	0.67
Other	0.93 (0.67–1.27)	0.63	1.08 (0.66–1.75)	0.77	0.98 (0.52–1.86)	0.96
**Hepatitis B**						
Neg/Not Tested	1.00		1.00		1.00	
Positive	1.05 (0.59–1.84)	0.88	1.21 (0.88–1.66)	0.24	1.14 (0.68–1.9)	0.62
**Hepatitis C**						
Neg/Not Tested	1.00		1.00		1.00	
Positive	0.63 (0.4–0.97)	0.04	0.87 (0.53–1.41)	0.56	0.65 (0.39–1.11)	0.12
**Year of ART Initiation**						
1996–2004	1.00		1.00		1.00	
2005–2011	0.81 (0.64–1.02)	0.08	0.7 (0.54–0.91)	0.01	0.62 (0.48–0.81)	<0.001
No	1.00		1.00		1.00	
Yes	1.43 (1.02–2.01)	0.04	0.97 (0.77–1.21)	0.77	1.15 (0.88–1.5)	0.32
**CD4 cell count at ART initiation (cells/µL)** *						
0–199	1.00		1.00		1.00	
200–350	1 (0.75–1.35)	0.99	1.28 (0.92–1.76)	0.14	1.19 (0.86–1.64)	0.29
350–500	1.1 (0.78–1.55)	0.60	1.45 (0.93–2.25)	0.10	0.61 (0.19–1.93)	0.40
>500	0.99 (0.68–1.45)	0.97	1.86 (1–3.45)	0.05	1.77 (0.72–4.36)	0.21
Unknown	1.12 (0.63–2.01)	0.69	1.1 (0.79–1.54)	0.58	1.24 (0.83–1.85)	0.29
**RNA–VL at ART initiation (copies/ml)** *						
1–10^4^	1.00		1.00		1.00	
10^4^–10^6^	0.76 (0.52–1.11)	0.15	0.71 (0.44–1.17)	0.18	1.59 (0.54–4.72)	0.40
>10^6^	0.81 (0.55–1.17)	0.26	0.78 (0.49–1.25)	0.31	2.24 (0.8–6.27)	0.13
Unknown	0.79 (0.43–1.44)	0.44	1.01 (0.62–1.64)	0.96	1.46 (0.54–3.99)	0.46

Hazard ratios calculated from multivariable model including adjustment for site resourcing.

* Closest record to ART initiation within a window of 6 month prior to ART and 1 week following ART initiation.

**Figure 3:**
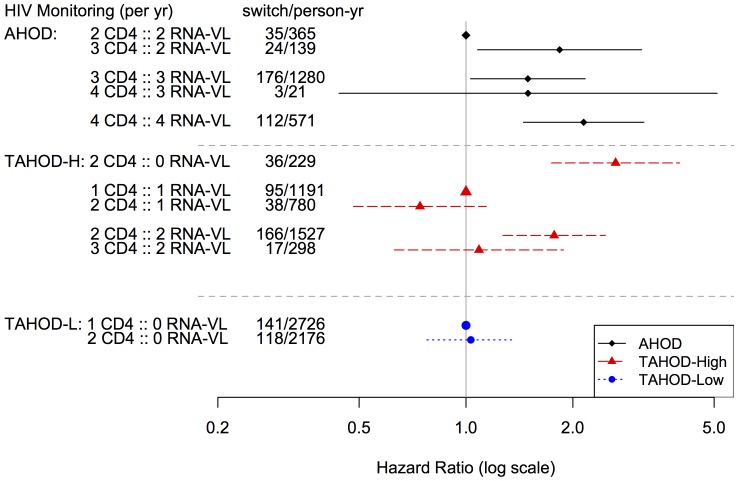
Multivariable adjusted associations for site resourcing and time to major first-line ART modification. Income group-specific models are adjusted for age, sex, exposure, HBV, HCV, pre-ART AIDS illness, pre-ART CD4, pre-ART RNA-VL and year of ART initiation.

Figure 3 plots multivariable model estimate hazard ratios for the site resourcing covariate by income group (adjusted for factors listed above). In AHOD, the relative base was two CD4 counts and two RNA-VLs per year (e.g. monitored twice-yearly). In AHOD, sites that monitored on average four-times per year corresponded with an 2-fold increase in the rate of major first-line modification. Similarly for TAHOD-High, relative to once-yearly monitoring (both CD4 counts and RNA-VL), sites that monitor twice-yearly demonstrated an increase in rate of first-line modifications by a factor of 1.8. No sites in TAHOD-Low monitored one-yearly CD4 count and RNA-VL concurrently. There were no differences in modifications to first-line ART for patients who had one or two CD4 count measurements per year.

## Discussion

In the Asia-Pacific region, the overall rate of major modification to first-line ART was associated with country income and HIV monitoring levels, particularly RNA-VL. Low- to lower middle-income countries typically had lower rates of major modification to first-line ART by a factor of 0.36 (0.31–0.41) relative to Australia. We found little evidence of significant associations between treatment modification and standard patient characteristics. By evaluating rates of major modifications and associations with a proxy indicator for site resourcing, we showed that there is a relationship between the number of RNA-VL tests and major modification to first-line ART. In AHOD, relative to sites that monitor twice-yearly, quarterly monitoring corresponded with an approximate doubling of the rate of major modification. We saw a similar pattern in TAHOD-High, where relative to sites that monitored once-yearly, a doubling of monitoring to twice-yearly corresponded to just below a doubling of major modifications. We were unable to evaluate this association in TAHOD-Low as no sites performed at least once-yearly concurrent monitoring of CD4 count and RNA-VL. Within the lower income sites, an increase of CD4 monitoring from once- to twice-yearly monitoring did not result in an increase in major modification made to first-line ART.

Given that one goal of HIV monitoring is to provide guidance on the optimal time to switch ART, it is unsurprising that increasing the frequency of RNA-VL monitoring also increases the rate of major modifications made to first-line ART. We speculate that regular and frequent point-of-care visits lead to continual dialogue and discussion of patient experiences and concerns about their wellbeing including review and discussion of all test results. Modification of ART may also be on the basis of reporting of adverse events, barriers to adherence, observed toxicity on routine test results (e.g. reduced estimated glomerular filtration rate, subclinical but biochemical hepatitis, anemia, etc.) leading to more treatment modifications aimed at maximising benefit and minimising adverse events. Figure 2 collectively outlines this point. The rate of treatment modification due to treatment failure is relatively low and similar across all income groups. However the rate of treatment modification reported due to adverse events/toxicity (or patient/physician decisions) is much higher in higher income countries compared to lower income countries. It is unlikely that the ART regimens distributed in lower income countries are less toxic or better tolerated than their higher income counterparts. Therefore we believe this data indicates that higher income countries have additional flexibility to make treatment modifications on varying levels of reported adverse events/toxicity or by choice of the patient or at the discretion of the treating physician.

It still remains unclear if access to all available ARVs and quarterly RNA-VL monitoring actually increases or decreases the number of unnecessary treatment modifications. An analysis by the EuroSIDA group found that the risk of virological failure following one year of well-tolerated fully suppressive ART is low [19]. Patients in that analysis were monitored quarterly and the authors concluded that twice-yearly monitoring could be an effective monitoring schedule for stable patients. To date there are few randomised clinical trial or cohort data evaluating the optimal timing of HIV disease monitoring that minimises cost and maximises treatment outcomes in high resource settings [20]. A cost-effectiveness analysis based on mathematical modelling assessed multiple HIV monitoring strategies in Southern Africa. The authors concluded that monitoring RNA-VL and CD4 count quarterly over twice-yearly has only a modest effect on additional life expectancy and contributes to a small reduction in opportunistic diseases [21].

Our study does not report mortality or treatment outcomes (clinical, immunological and virological) between the different income groups. Although not directly comparable due to differing analysis populations, treatment outcome differences in the Asia-Pacific have been reported elsewhere [22]–[25]. Briefly, Egger et al reported a lower absolute mean difference of in mean CD4 cell counts [−47 to −7 cell/µL] in TAHOD compared to AHOD [23]. A follow-up study by Achhra et al concluded that this mean CD4 cell count difference translated to minimal clinical significance in terms of mortality and new AIDS-defining illness [22]. A comprehensive analysis of AIDS-related and non-AIDS-related mortality in AHOD and TAHOD also reported little evidence of differing hazard ratios of mortality in TAHOD-High and TAHOD-Low relative to AHOD [24]. However, in a TAHOD-specific study, Oyomopito et al reported less favorable treatment outcomes for sites that reported less than once-yearly RNA-VL testing compared to those sites that monitored RNA-VL at least once-yearly [25]. In aggregate these finding suggest that there are minimal population differences in treatment outcomes between AHOD and TAHOD. Collectively these results are consistent with a study conducted by the Swiss HIV Cohort Study (SHCS) and the International Epidemiologic Databases to Evaluate AIDS in Southern Africa (IeDEA-SA) group that directly compared HIV treatment outcomes between a high-income country (Switzerland) and a low-income country (South Africa) [26]. The investigators concluded that although patients switched treatment much more often in Switzerland, there were minimal differences between longer-term treatment outcomes.

Our results are consistent with studies that have previously evaluated factors associated with switching from first-line ART to second-line regimens in both resource-rich and resource-limited settings. Although we cannot compare our results directly due to differing analysis designs- including reasons for switching algorithms and importantly, regional differences in antiretroviral treatment guidelines. These studies have found few factors associated with switching from first-line ART. Of the large observational cohorts evaluating switching in resource-limited settings, a large multi-country cohort analysis from the ART-LINC group showed an increasing risk association between switching to second-line ART (for any reason) and low pre-ART CD4 cell count (<150 cells/µL) and earlier time periods of ART initiation [27]. Similar associations, low pre-ART CD4 and earlier time-periods of ART initiation were also found in Sub-Saharan Africa [28].

There are limitations to our analysis. First, although we have designed an analysis based largely on prospective treatment records from a population of patients receiving routine care at multiple sites throughout the Asia-Pacific region, we are unable to quantify selection bias due to the observational nature of the data. Second, in our analysis we have used World Bank-defined criteria to categorise countries into high/middle/low-income groupings based on gross national income. Clearly, economic prosperity or disparity change over time, and we note that our income groupings and the associated results are specific to the observed time period. Furthermore, we note that the ART treatment guidelines have changed overtime. Although we have attempted to address these differences through adjusting for year of ART initiation, we are unable to quantify any remaining impact on our result for these treatment guideline changes. Third, our association analysis endpoint (Table 4, Figure 3) is composite and captures all major modifications to first-line ART regardless of reason. Although we have recorded reasons for modification, we are hesitant to differentiate the analysis (e.g using a competing risk framework) based on this variable. Reasons for treatment modification are complex due to co-dependence, where adverse events influence adherence, which in turn leads to treatment failure. Furthermore, there are a large number of unreported reasons for modification in AHOD and exclusion of these events would likely introduce bias due-to missing information. Finally, we are unable to evaluate any site-specific bias in the proxy indicator variable for site resourcing. A given resourcing level (e.g. 2CD4::1RNA-VL) may actually correspond to only a single site within the income group, thus limiting interpretation of site resourcing categories with infrequent events or observed person-years.

In summary, we found that low-income countries tended to have lower rates of major modifications made to first-line ART compared to higher income-countries, where increased HIV monitoring led to increased modifications of first-line ART. The clinical significance of this finding at an individual patient level is unknown. Given the current need for life-long therapy in the management of HIV, further research evaluating the optimal monitoring schedule which balances overall cost and favorable treatment outcomes would be of value to both resource-rich and resource-limited countries.
